# Systematic evaluation of chromatin immunoprecipitation sequencing to study histone occupancy in dormancy transitions of grapevine buds

**DOI:** 10.1093/treephys/tpac146

**Published:** 2023-01-13

**Authors:** Dina Hermawaty, Jonathan Cahn, Ryan Lister, Michael J Considine

**Affiliations:** The UWA Institute of Agriculture, The University of Western Australia, M082/35 Striling Hwy, Perth, WA 6009, Australia; ARC Centre of Excellence in Plant Energy Biology, The University of Western Australia, M310/35 Striling Hwy, Perth, WA 6009, Australia; ARC Centre of Excellence in Plant Energy Biology, The University of Western Australia, M310/35 Striling Hwy, Perth, WA 6009, Australia; The UWA Institute of Agriculture, The University of Western Australia, M082/35 Striling Hwy, Perth, WA 6009, Australia; ARC Centre of Excellence in Plant Energy Biology, The University of Western Australia, M310/35 Striling Hwy, Perth, WA 6009, Australia; Horticulture and Irrigated Agriculture, Department of Primary Industries and Regional Development, 1 Nash St, Perth, 6000, Australia

**Keywords:** DNA sequencing, DNA–protein interactions, epigenetic, perennial plant, woody tissues

## Abstract

The regulation of DNA accessibility by histone modification has emerged as a paradigm of developmental and environmental programming. Chromatin immunoprecipitation followed by sequencing (ChIP-seq) is a versatile tool to investigate in vivo protein–DNA interaction and has enabled advances in mechanistic understanding of physiologies. The technique has been successfully demonstrated in several plant species and tissues; however, it has remained challenging in woody tissues, in particular complex structures such as perennating buds. Here we developed a ChIP method specifically for mature dormant buds of grapevine (*Vitis vinifera* cv. Cabernet Sauvignon). Each step of the protocol was systematically optimized, including crosslinking, chromatin extraction, sonication and antibody validation. Analysis of histone H3-enriched DNA was performed to evaluate the success of the protocol and identify occupancy of histone H3 along grapevine bud chromatin. To our best knowledge, this is the first ChIP experiment protocol optimized for the grapevine bud system.

## Introduction

Chromatin immunoprecipitation (ChIP) enables the study of DNA–protein interactions and has become a method of choice for studying the trans-regulation of gene expression, as well as post-translation histone modification. The technique was developed following a report that demonstrated reversible crosslinking of nucleosome–DNA by formaldehyde (Jackson 1978, Klockenbusch et al. 2012). In combination with several DNA assay techniques, such as southern blotting (Solomon et al. 1988, Orlando et al. 1997), polymerase chain reaction (Hecht et al. 1996), microarray (Iyer et al. 2001) and sequencing (Johnson et al. 2007), the DNA sequence associated with the protein of interest may be identified. Forty years after its development, ChIP has been extensively used to study epigenetic regulation in animal and yeast cells, but only recently applied in plants (Johnson et al. 2001, Wang et al. 2002). The delay in uptake of ChIP in plant science was due to several impediments, particularly: (i) a large amount of tissue is typically needed, (ii) the presence of cell walls required vigorous physical disruption therefore sample loss during the process is unavoidable and resulted in low DNA yield, (iii) co-extraction and precipitation of interfering compounds often problematic for downstream analysis such as PCR/qPCR and library preparation, (iv) limited availability of ChIP-grade antibodies specific for plant cells often leading to a false-negative signal and (v) the comprehensive ENCODE guidelines for model biological system is not always applicable for plant biology research.

The intriguing and complex regulation of plant developmental processes, as a response to environmental stimuli, has driven many studies on gene expression regulation in an epigenetic context. The vernalization requirement for flowering of Arabidopsis is established by the flowering repressor FLOWERING LOCUS C (FLC), whereby chilling-dependent histone modification of the *FLC* locus represses transcription and hence enables flowering (Michaels and Amasino 1999, Helliwell et al. 2006). As histones are widely conserved and several commercial antibodies available, ChIP has been successfully applied to non-model plant studies also, including dormancy in perennial buds (Leida et al. 2012, de la Fuente et al. 2015, Saito et al. 2015, Vimont et al. 2020). To date, protocols guiding ChIP experiments in plant systems, such as Arabidopsis (Saleh et al. 2008), tomato (Ricardi et al. 2010), maize (Haring et al. 2007) followed by DNA microarray hybridization (Reimer and Turck 2010) or sequencing (Kaufmann et al. 2010) have been published. However, the variables amongst these studies illustrate the need to tailor conditions to each experiment, and in particular each tissue type (Park 2009, Landt et al. 2012). As such, protocols established for soft tissues such as leaves (Saleh et al. 2008) or seedlings (Ricardi et al. 2010) are likely to be ineffective for seed (Haque et al. 2018) or wood forming tissues (Li et al. 2014*b*). Further, metastudies have shown that even commercially available ChIP-grade antibodies may fail control tests for specificity (Egelhofer et al. 2011). In some cases, batch information of these validation steps is available either on the ENCODE Project website (Davis et al. 2017) or subsites (Egelhofer et al. 2011) or via the manufacturer. Alternatively, the antibody/s must be validated before commencing ChIP experiment (Landt et al. 2012). Procedures and criteria for antibody validation have been well-outlined by members of the ENCODE Project, however, these were specifically developed for animal tissues, and hence neglect for example the additional constraints of working with plant cell walls and particularly lignified tissues.

**Figure 1:**
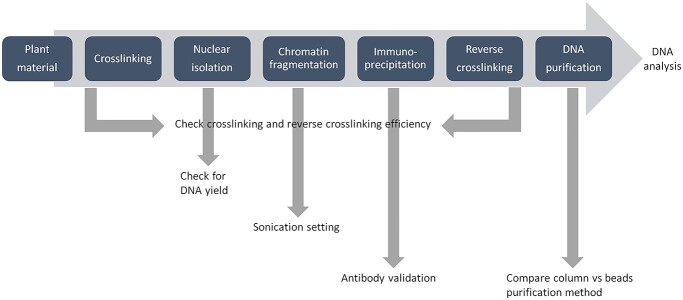
Chromatin immunoprecipitation workflow with checkpoints indicated by the gray arrow.

The ChIP workflow is summarized in Figure 1. In brief, the interaction of protein and DNA (collectively known as chromatin) is crosslinked in vivo by incubation of tissue in formaldehyde solution. The crosslinked chromatin is then fragmented by sonication, which breaks the chromatin into short fragments that are suitable for the subsequent processes. The protein–DNA complex is co-precipitated using antibody allowing selective precipitation of DNA that interacts with protein of interest. The precipitated DNA is released from the protein by reverse crosslinking and subsequently assayed to identify the sequence. Each step in the ChIP procedure is prone to high variability; for example, sonication must be titrated to ensure the optimal size of chromatin while preventing damage. Similarly, for crosslinking, insufficient crosslinking could cause poor preservation of chromatin and its associated protein and significantly reduce the yield of DNA at the end of the immunoprecipitation process (Orlando 2000). Alternatively, excessive crosslinking can make the chromatin brittle and prevent efficient reversibility of the crosslinking at subsequent steps. Therefore, optimization needs to be systematic in order that the method is robust and reproducible, yielding maximum enriched DNA (Figure 1, arrow).

The experimental system used in this study is an adaptation of the ChIP-seq workflow published by the ENCODE and monENCODE consortia (Landt et al. 2012). For more than a decade, the consortia have done extensive ChIP experiments conducting more than 140 experimental variables in more than 100 cell types. The guidelines aim to provide a standardized protocol for conducting ChIP seq works that enables data comparison across multiple studies. However, the ENCODE guidelines were developed from model animal cells or tissue experimental setup. Understanding the role of epigenetics in plant systems requires transfer of knowledge and methods from model research and transforming the tools specifically for studying a non-model species, in this case, a woody plant. This has been proved challenging given the physical and molecular characteristics of plant cells. The ChIP protocol we describe is a modified procedure optimized for wood-forming xylem tissue developed by Li et al. (2014*b*), which provides a guide to cope with the difficulties of working with woody tissue. Systematic optimization was performed according to ENCODE guidelines for ChIP experiment (Landt et al. 2012) and other recommendations from previously published ChIP protocols with plant tissue (Haring et al. 2007, Ricardi et al. 2010, Song et al. 2016). Chromatin immunoprecipitation was performed using a ChIP kit manufactured by Abcam to eliminate washing steps after immunoprecipitation which often contribute to the loss of enriched DNA. Finally, we performed, DNA sequencing and identified the gene that was occupied by histone H3 protein.

## Materials and equipment

### Plant material and treatment

**Figure 2:**
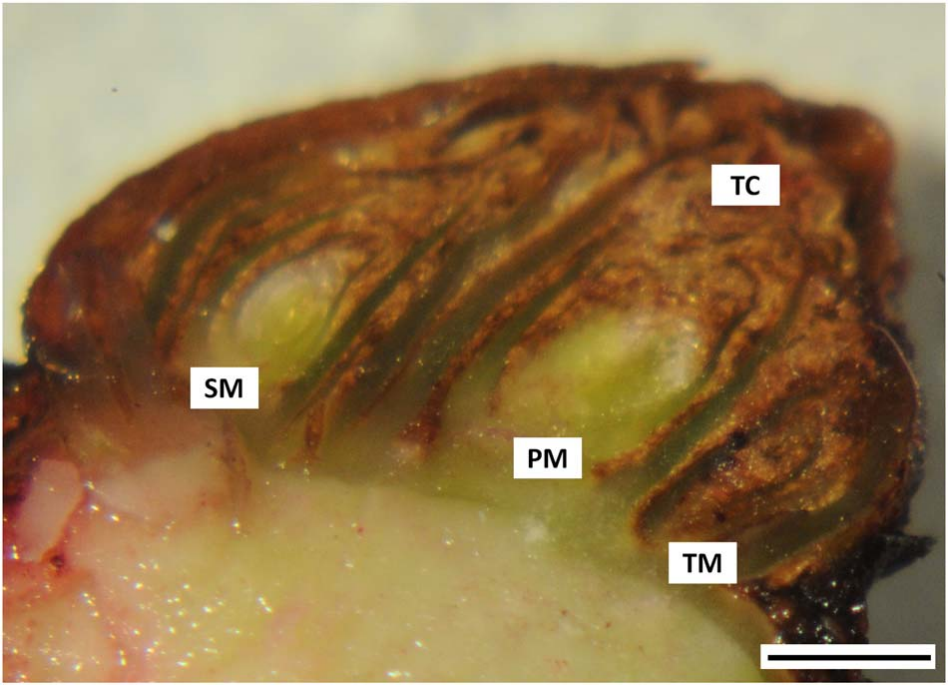
Anatomy of grapevine axillary bud. Trichome (TC) hairs are shown as the brown-color structures that surround the green tissue (PM, primary meristem; SM, secondary meristem; TM, tertiary meristem) of the axillary bud. Scale bar = 1 mm.

The mature dormant buds of *Vitis vinifera* (L.) cv. Cabernet Sauvignon (Figure 2) were collected from a vineyard in Margaret River, Australia (34°S, 115°E) on March, May and August. Each cutting consisted of four mature buds from nodes 4 to 7. The canes were immediately transported to the lab in damp newsprint in an insulated box and stored at 22 °C for up to 24 h. Treatment with hydrogen cyanamide (H_2_CN_2_; Sigma-Aldrich #187364) was done to buds harvested in March by submerging the node into 1.25% (w/v) [300 mM] H_2_CN_2_ for 30 s. Control buds were treated in the same manner with water (W). The explants were then stored in the dark for 24 h at room temperature before being crosslinked.

### Chromatin immunoprecipitation

The chromatin immunoprecipitation procedure consists of six main steps, (i) crosslinking, (ii) nuclear isolation, (iii) DNA fragmentation, (iv) chromatin immunoprecipitation, (v) reverse crosslinking and (vi) purification/recovery of immunoprecipitated DNA. Briefly, collected bud tissue was excised from the cane, dissected in half and immediately immersed in crosslinking buffer before cycled vacuum was applied to facilitate infiltration into bud tissue. For nuclear isolation, 50 ml buffer 1 was added to 10 g of ground crosslinked buds and homogenized using ULTRA-TURRAX homogenizer. The crude homogenate was passed through three layers of Miracloth and centrifuged at 2880*g* for 10 min at 4 °C. The pellet containing nuclear was resuspended in buffer 2, centrifuged at 16,000*g* for 10 min at 4 °C, and pellet was then resuspended in buffer 3. Intact nuclear was extracted by layering the suspension on top of a cushion of buffer 3, then centrifuged at 16,000*g* for 60 min at 4 °C. Clean nuclei pellet was then resuspended in lysis buffer and sheared into fragments of 200–400 bp using a Covaris S220 focus-ultrasonicator. Chromatin immunoprecipitation and reverse crosslinking were performed following procedure by the Abcam ChIP kit plant (Abcam, cat. no. ab117137) with some modification. Lastly, immunoprecipitated DNA was purified using Agentcourt AMPure XP beads (Beckman Coulter Life Science, USA, cat. no. A63881) adjusted to recover DNA fragment larger than 100 bp. Detailed step-by-step procedure, important notes, and checkpoints for chromatin immunoprecipitation are provided as Method S1, available as Supplementary data at *Tree Physiology* Online. Nuclei extraction and immunoprecipitation were performed in three biological replicates.

### Sequencing

For sequencing, DNA enriched by histone H3 immunoprecipitation from three biological replicates were pooled to make 1 library each for the sample collected in March, May, August, and buds treated with H_2_CN_2_; the low amount of immunoprecipitated DNA resulting from ChIP with modified histone H3 precluded sequencing biological replicates. The library was constructed using NEBNext^®^ Ultra™ II DNA Library Prep Kit for Illumina^®^ following manufacturer’s low-input ChIP-seq protocol (Method S2, available as Supplementary data at *Tree Physiology* Online). The library for input and histone H3-enriched DNA each from March (water- and H_2_CN_2_-treated), May and August samples were sequenced at Genewiz Genomics Centre (Suzhou, China) as pair-end (PE), 150 bp for an average of 40 million of reads per sample. Raw reads were trimmed for quality and adaptors using Trimmomatic v0.39 (Bolger et al. 2014). Post-trimming read quality was assessed using FastQC and results were aggregated using MultiQC (Ewels et al. 2016). The remaining reads were mapped to the 12X V1 *Vitis vinifera* PN40024 reference genome (Jaillon et al. 2007) using the Burrows–Wheeler Aligner (BWA) (Li and Durbin 2009). Peak calling was conducted using MACS2 software version 2.1.0 (https://github.com/taoliu/MACS) with cut off *q*-value < 0.05. The annotatePeaks.pl algorithm of the HOMER (Hypergeometric Optimization of Motif EnRichment) suite of tools (Heinz et al. 2010) was used to annotate the peaks. DeepTools (Ramírez et al. 2016) was used to process the mapped reads data for creating normalized coverage files in standard bedGraph and bigWig file formats to visualize and compare different files. Functional category enrichment was performed for genes that were enriched by histone H3 using topGO package following a grapevine-specific functional classification of 12X V1 predicted transcript (Grimplet et al. 2012) with modification according to the GO database (Ashburner et al. 2000). A Fisher’s exact test (*P* < 0.05) was carried out in topGO to compare each study list with the list of total non-redundant transcript housed in grapevine 12X V1 gene predictions (Grimplet et al. 2012). The gene ontology (GO) terms were further simplified using REVIGO allowing similarity of 0.5 (Supek et al. 2011).

## Results

### Crosslinking by vacuum infiltration

**Figure 3:**
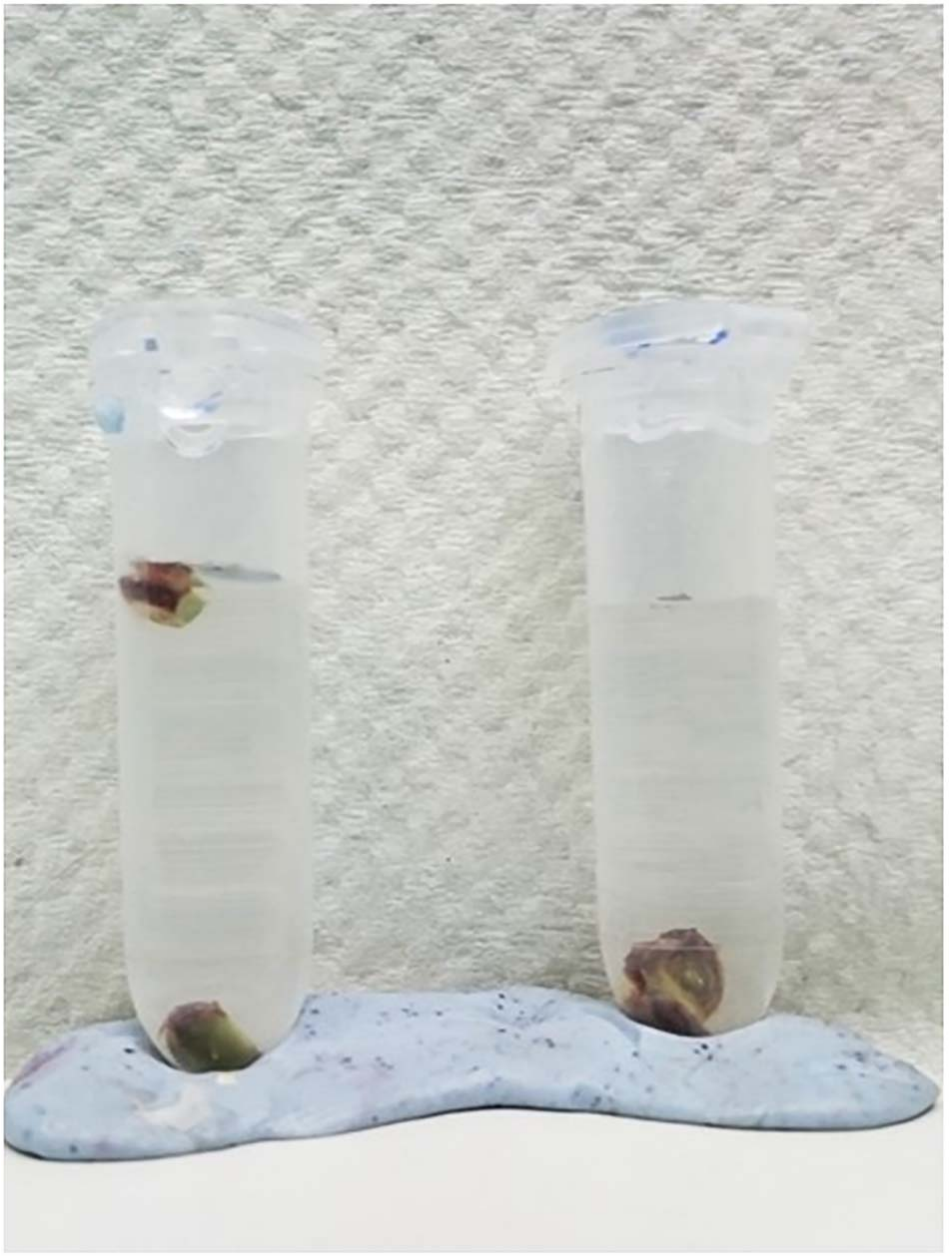
Fixative infiltration optimization. Buds were cut into half before immersed into the fixative solution. Cycled vacuum was applied by performing three cycles of 5 min vacuum, release and mix at room temperature. An efficient penetration of the fixative was evident after vacuum indicated by increasing of the bud density that causes buds sunk into the bottom of the tube. Cycled vacuum method also allows short crosslinking process that is preferred for ChIP analysis.

Infiltration with 15 min cycled vacuum (5 min vacuum/release/ mix × 3) and without vacuum was compared to determine a suitable infiltration method for grapevine buds. Complete infiltration was indicated by the movement of buds to the bottom of the tube as the bud density became higher after infiltration of crosslinking buffer (Figure 3).

**Figure 4:**
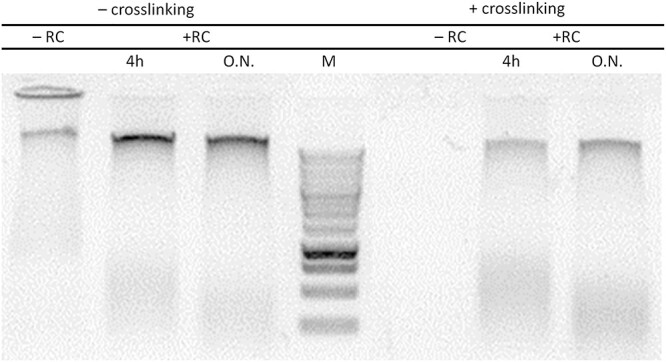
Crosslinking and reverse crosslinking efficiency. Nuclear extract was prepared from grapevine buds without (−) crosslinking and with (+) crosslinking treatment. Grapevine buds were crosslinked in crosslinking buffer containing 1% formaldehyde for 15 min (3 × 5 min vacuum cycles) at room temperature. The sample was reverse crosslinking (+RC) for 4 h and over the night (O/N) or not (–RC). DNA was purified using phenol/chloroform extraction followed by ethanol precipitation. DNA recovery was compared between samples with and without crosslinking.

The phenol:chloroform:isoamyl alcohol (PCI) solution separates nucleic acid and protein based on its solubility in the solvents, nuclei acid is soluble in aqueous phase while protein in organic phase. Excessive crosslinking or ineffective reverse crosslinking will retain interaction between DNA and protein and therefore reduce the amount of DNA in the aqueous phase because the protein–DNA complex will be soluble in the organic phase instead. Crosslinking efficiency of our protocol was then assessed by comparing amount of DNA in the aqueous phase from crosslinked and non-crosslinked bud, treated with or without reverse crosslinking. In non-crosslinked bud (Figure 4, lanes 1–3), DNA was soluble in the aqueous phase with or without reverse crosslinking treatment. In contrast, when crosslinking was performed, DNA can only be recovered from the aqueous phase if a reverse crosslinking procedure was conducted (Figure 4, lane 6). The overnight reverse crosslinking procedure can be done as an alternative to a shorter duration without affecting DNA recovery (Figure 4, lane 7). Absence of DNA at lane 5 confirmed the successful crosslinking procedure that maintains the protein–DNA interaction, while presence of DNA at lanes 6–7 demonstrates efficiency of our crosslinking allowing release of DNA from protein.

### Chromatin yield and nuclei integrity

**Figure 5:**
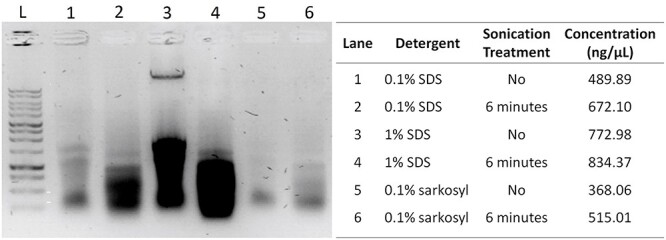
The combination of type and concentration of detergent in the lysis buffer and application of sonication resulted in a different yield of DNA. L: 1Kb DNA ladder (Promega #G5711) in 1% agarose gel, DNA quantification was performed using a NanoDrop 1000.

**Figure 6:**
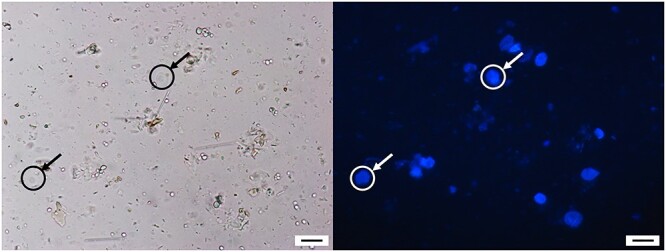
Nuclei integrity assessment by examination under a microscope. DAPI stain DNA specifically at the A-T rich region and will emit blue fluorescence light that can be observed using an epiluminescence microscope. The image was taken using DAPI filter (exciter filter BP 365/12, chromatic beam splitter FT 395, and barrier filter LP 397). Bar = 5 μm.

Disruption of antigen–antibody interaction was mainly avoided in most ChIP protocols by using 1% SDS in lysis buffer and further diluting the chromatin suspension after DNA fragmentation to reduce the SDS concentration to 0.1%. We obtained the highest DNA yield using 1% SDS (Figure 5, lanes 3–4); however, a considerable increase in DNA yield was observed after application of 6 min of sonication in sample lysed using low detergent concentration (Figure 5, lanes 1–2 and 5–6). An aliquot of 6 min sonicated nuclei suspension (see Method S1 step 16, available as Supplementary data at *Tree Physiology* Online) was stained with DAPI (1 μg ml^−1^) and subjected to microscopic observation to assess the integrity of nuclei. The micrograph showed a uniform, intact and well-separated nucleus (Figure 6).

### DNA fragmentation

**Figure 7:**
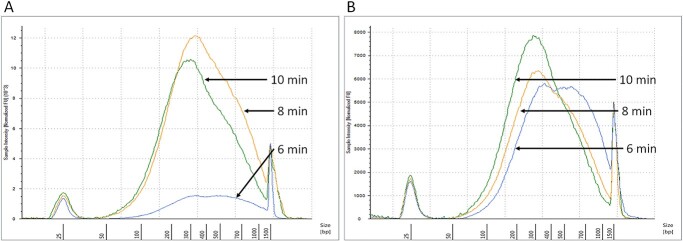
Optimization of chromatin fragmentation. Chromatin fragmentation was optimized to obtain suitable DNA fragment size for ChIP-seq, 200–400 bp. Chromatin extracted using 0.1% (A) and 1% SDS (B) were sonicated for 6 (blue), 8 (yellow) and 10 (green) minutes. Distribution of DNA fragment size was analyzed using Agilent bioanalyzer. Accumulation of smaller DNA fragment was linear to sonication duration with suitable average fragment size was obtained after 8 min, and more accumulation of fragment size from 200 to 400 bp observed after 10 min sonication.

A sonicator setting to produce an average of 300 bp fragment was used, following the default setting provided by Covaris S220 Focused-ultrasonicator manufacture. In general, short DNA fragments were gradually accumulated as sonication duration increased (Figure 7). After 8 min of sonication, the average fragment size was within the 200–400 bp range. Increasing the duration of sonication to 10 min resulted in greater accumulation of DNA fragments in the 200–400 bp range without causing further fragmentation of the short DNA.

### Yield of immunoprecipitated-DNA

Three different methods to purify the immunoprecipitated-DNA were tested in which the lowest DNA recovery was produced by column purification method while the paramagnetic beads (AMPure XP) resulted the highest DNA yield (Table 1). Therefore, we substitute the column purification from the original Abcam ChIP kit protocol with purification using AMPure XP beads (see Method S1 step 35, available as Supplementary data at *Tree Physiology* Online). Generally, we enriched 10% of input DNA by histone H3 and only 1% by modified histone H3 antibody using 5- or 10-g buds to perform ChIP experiment for three antibodies (Table 2). The amount of enriched-DNA from the modified histone H3 was considered too low for protocol validation using quantitative polymerase chain reaction (ChIP-qPCR) or conventional library construction for several reasons. First, our qPCR titration experiment showed that the lowest DNA concentration that can be detected by the qPCR machine should be no less than 0.1 ng μl^−1^ (Table S1, available as Supplementary data at *Tree Physiology* Online). Second, there was no available positive control DNA target region for native- or modified-histone H3 in grapevine that could be used for ChIP protocol validation by qPCR. Lastly, library construction results were highly variable when DNA template was less than five ng.

**Table 1:** The yield of DNA using three different purification method.

Purification method	DNA conc.^1^	DNA yield
	(ng μl^−1^)	s(μg g^−1^)
Abcam kit column purification	0.71	0.14
Phenol/Chloroform/Isoamyl alcohol	6.56	1.31
AMPure XP beads	31.53	6.31

^1^DNA concentration was measured using Qubit fluorometer.

**Table 2:** The average yield of input and ChIP-enriched DNA resulted from ChIP experiment using 5 and 10 g of bud tissue for chromatin extraction (*n* = 3).

Sample name	5 g	10 g
	Yield (ng)	Yield (ng)
MH_input	274.8	398.7
MH_histone H3	32.0	29.9
MH_H3K4me3	1.1	3.2
MH_H3K27me3	6.2	3.2
MW_input	305.6	412.2
MW_histone H3	28.2	36.8
MW_H3K4me3	1.3	2.8
MW_H3K27me3	1.9	3.5
May_input	244.7	305.3
May_histone H3	19.6	24.5
May_H3K4me3	1.1	2.4
May_H3K27me3	2.8	2.9
Aug_input	264.4	285.7
Aug_histone H3	16.7	13.4
Aug_H3K4me3	0.9	2.5
Aug_H3K27me3	1.4	2.3

Abbreviations: MH, March H_2_CN_2_-treated buds, MW, March water-treated buds. Note: On May and August buds were only treated with water.

### Antibody validation

Antibody recognition in grapevine buds was confirmed by Western blot analysis of grapevine buds nuclear extract recognizing a ~17 kDa band corresponding to predicted molecular weight of histone H3 and H3K4me3. The ImageJ software was used to estimate the signal intensity produced by each antibody (data not shown).

**Figure 8:**
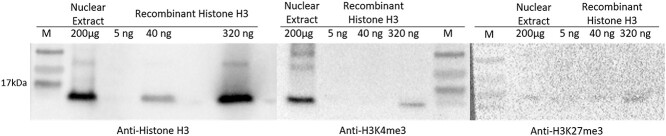
Representative western blotting assay for ChIP-antibody validation. Three antibodies used in ChIP assay were used for immunoblotting against nuclear extract prepared from grapevine buds and recombinant histone H3 at the concentration indicated in the image above. All antibodies were considered to pass validation test with detection of histone H3 protein and negative signal in H3K4me3 and H3K27me3 protein at 40 ng.

Immunoblot against anti-histone H3 showed detection limit of the antibody is around 40 ng and 200 μg nuclear extract containing a little less than 320 ng histone H3 protein (Figure 8, panel 1). Anti-H3K4me3 passed the test showing absence of signal against 40 ng recombinant histone H3 protein (unmodified) (Figure 8, panel 2). A false-positive signal observed against 320 ng recombinant histone H3 protein was observed; however, the intensity of the signal is no more than one-tenth the nuclear signal. No signal was observed in the nuclear extract tested against the anti-H3K27me3. We recognize that the lack of signal did not definitively indicate failure of the antibody, as this may result from low abundance of the modified histone in the tissue used for this test (Figure 8, panel 3).

### Histone H3 occupancy

Due to the poor yield of ChIP DNA, three biological replicates of each treatment condition were pooled for sequencing. We generated an average 40 million 150 bp paired end reads from each of the histone H3-enriched and input DNA libraries of water-treated March (3W), May (5W), August (8W) and H_2_CN_2_-treated March buds (3H) buds. Although statistical comparisons cannot be made, it is worthwhile describing the trends.

**Figure 9:**
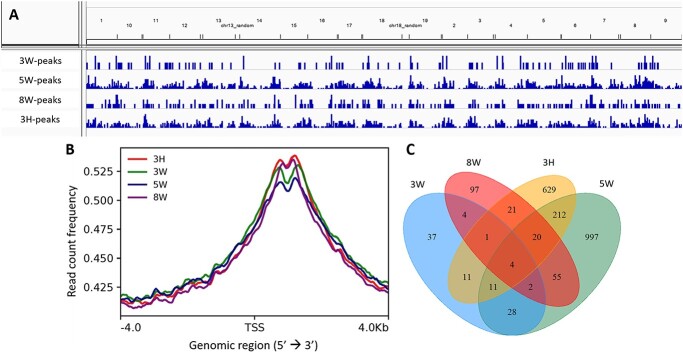
Chromatin immunoprecipitated-DNA peak analysis. (A) Distribution of histone H3 peaks along *Vitis vinifera* genome at each condition. (B) The average profile of ChIP peak binding at the TSS region showing read count frequency range from −4000 to 4000 bp. (C) The Venn diagram of genes identified downstream TSS from buds collected in March, May, August treated with water and March buds treated with H_2_CN_2_.

About 90% of reads remained following trimming and were mapped uniquely to the grapevine reference genome (Table S2, available as Supplementary data at *Tree Physiology* Online). Here, we showed a peak binding distribution of histone H3 at regions 4000 bp up- and down-stream of transcription start site (TSS) in each condition. The highest occupancy was observed in the genic (exon, intron or intergenic) region (Figure 9). ChIP peak calling analysis identified different peaks at each condition, with the highest found in the May and H_2_CN_2_-treated March conditions and the lowest in the water-treated March and August conditions (Figure 9).

We further explore this preliminary data to gain insight into the region associated with histone H3 protein. Earlier studies comparing nucleosome occupancy and gene expression in *Arabidopsis* showed that genes with higher transcript abundance tend to be relatively unoccupied by nucleosomes at the promoter area but relatively enriched in the genic region immediately downstream of the TSS (Valouev et al. 2011, Li et al. 2014*a*). Based on this information, we focus our exploration on genes that may be expressed at each time of harvesting and therefore gene identifiers in the genic region (not the promoter region). Annotation of the DNA associated with the histone H3 peaks in the genic region identified 129, 1691, 291 and 1207 genes for the 3W, 5W, 8W and 3H conditions (Table S3, available as Supplementary data at *Tree Physiology* Online). The Venn diagram analysis shows that only a few genes were commonly identified across samples, except for the May condition (5W) and March H_2_CN_2_ treatment (3H), with 247 common genes (Figure 9). Next, gene ontology analysis was performed to identify the biological processes that are associated with the associated genic region. Relatively few biological processes were enriched in water-treated March and August condition buds by comparison with the May condition and buds treated with H_2_CN_2_. Categories related to meristem developmental state were enriched in water-treated March and May conditions, represented by embryonic morphogenesis (GO:0048598) in March and post-embryonic development (GO:0009791) in May. Meanwhile, the response to cold (GO:0009409) category was enriched coincident with prolonged exposure to cold in August. Enrichment of categories related to cell growth (GO:0016049) and cell differentiation (GO:0030154) was seen in H_2_CN_2_-treated buds (Table S4, available as Supplementary data at *Tree Physiology* Online). Lastly, we performed GO enrichment for the common gene identifiers found in May and H_2_CN_2_-treated buds to gain insight into biological processes that were commonly regulated when buds were released from their dormant state, either naturally by prolonged cold or forced by chemical application (Figure S1 and Table S5, available as Supplementary data at *Tree Physiology* Online). The results showed enrichment of categories related to response to starvation (GO:0042594), post-embryonic development (GO:0009791), and the regulation of phase transitions from vegetative to reproductive (GO:0048510) in both conditions. The genes associated with the enriched category were found to be involved in autophagy, flowering time, reactive oxygen species detoxification, sugar signaling, ABA-mediated signaling and pleiotropic responses (Table 3).

**Table 3:** Gene associated with enriched category of common gene found in May and H_2_CN_2_-treated buds.

Vv.ID	At.ID	Associated GO category	Functional.annotation	Note	Reference
VIT_17s0000g07160	AT5G61150	Response to abiotic stimulus, response to heat (vernalization response)	Vernalization independence 4 (VIP4)	Cold-independent regulator of flowering-time genes.	Zhang and Van Nocker (2002)
VIT_17s0000g09980	AT3G48430	Post-embryonic developmental, developmental process, histone modification	Relative of early flowering 6 (REF6)	Regulating flowering time through histone modification at flowering locus C (FLC) chromatin and demethylate histone 3 lysin 27.	Noh et al. (2004), Lu et al. (2011)
VIT_05s0124g00250	AT2G31650	Post-embryonic developmental, developmental process, histone modification	Histone-lysine N-methyltransferase ATX1	An Arabidopsis homolog of trithorax factor regulating flower organogenesis through histone 3 lysine 4 trimethylation.	Pien et al. (2008), Choi et al. (2014)
VIT_01s0011g02120	AT5G23150	Developmental process, regulation of timing of transition from vegetative to reproductive phase	Enhancer of AG-4 2 (HUA2)	Activate FLC expression and enhance AGMOUS function.	Chen and Meyerowitz (1999), Doyle et al. (2005)
VIT_02s0012g01930	AT1G32230	Post-embryonic developmental, developmental process, response to abiotic stimulus, response to superoxide.	Radical-induced cell death1 (RCD1)	Involved in stress-induced morphogenic response (SIMR) and maintaining meristematic fate by controlling redox balance.	Teotia and Lamb (2010), Brosche et al. (2014)
VIT_07s0104g00320	AT3G63080	Post-embryonic developmental, developmental process, response to stimulus.	Glutathione peroxidase 4	Reactive oxygen species detoxification process.	Milla et al. (2003)
VIT_04s0044g01750	AT2G17420	Post-embryonic developmental, developmental process, thioredoxin reduction (response to superoxide).	Thioredoxin reductase 2	Reactive oxygen species detoxification process.	Cha et al. (2015), Daloso et al. (2015)
VIT_14s0060g02380	AT3G62770	Response to starvation.	Autophagy 18 ATG18d	Required for autophagosome formation during nutrient deprivation or senescence and degradation of oxidase protein during oxidation stress.	Xiong et al. (2005, 2007)
VIT_05s0077g02310	AT4G15900	Post-embryonic developmental, sugar mediated signaling pathway.	PP1/PP2A phosphatases pleiotropic regulator PRL1	A nuclear WD-protein functions as a pleiotropic regulator of glucose and hormone responses during development in Arabidopsis.	Németh et al. (1998)
VIT_18s0001g06310	AT1G78290	Response to abiotic stimulus.	SnRK2–8	Involved in Abscisic Acid (ABA)-dependent growth by regulating expression of ABA insensitive 3 transcription factor.	Wu et al. (2019)

## Discussion

### Optimization conditions

#### Plant material

The amount of tissue used in ChIP experiment with plant tissue varies depending on tissue type. Several early studies used 100 g tissue per ChIP experiment (Ascenzi and Gantt 1999, Chua et al. 2001) but recent improvements have enabled efficient ChIP with 1–5 g, or 1 × 10^5^ purified nuclei (Gendrel et al. 2005, Deal and Henikoff 2011). The axillary buds of grapevine are heterogeneous organs consisting of multiple vegetative and reproductive meristems and leaves. The bud is covered in trichome hairs and consists of very little green tissue (Figure 10). Therefore, the low nuclei yield was expected. In this experiment, isolated chromatin from 10 g of buds was divided equally into four aliquots, each for immunoprecipitation using histone H3, H3K4me3, H3K27me3 and IgG antibodies. Although we established that there was no antibody cross-reactivity (discussed further in Antibody validation section), the amount of immunoprecipitated DNA recovered from the modified histone H3 is considered too low both for sequencing library preparation or quantitative PCR analysis using a gene of interest. Table 2 showed that the initial amount of DNA (input DNA) for each antibody reaction was around 300–400 ng. On average about 1% of DNA was recovered from immunoprecipitation using the modified histone H3 and 10% from the unmodified histone H3. Further, even after pooling three replicates into one when preparing the library for sequencing, only DNA recovered from immunoprecipitation using histone H3 was successfully used for library preparation (data not shown). Our experiment indicated that at the minimum, ChIP-seq library preparation requires at least 10 ng of DNA to generate a high quality library. Noting that the histone modified antibodies mainly recover about 1% of input DNA, we suggest that 400 buds (±10 g) were required for a ChIP experiment using one protein of interest (histone H3 or H3K4me3 or H3K27me3) and one control (histone H3 or IgG).

#### Crosslinking

Optimizing the incubation conditions for crosslinking is crucial for successful and efficient crosslinking (Orlando 2000). A short incubation duration for crosslinking is preferred in a ChIP experiment. Established protocol with yeast (Shivaswamy and Iyer 2007), alga (Strenkert et al. 2011), animal (Browne et al. 2014) or plant (Li et al. 2014*b*) cells usually apply 10–30 min incubation for crosslinking procedure. However, the hair-like structures inside buds create air spaces that could impede penetration of the crosslinking solution. The application of a vacuum cycle procedure was used here to change the pressure around the buds and remove entrapped air, thus allowing more efficient infiltration (Li et al. 2014*b*, Clode 2015). To test the efficiency of our vacuum infiltration technique, we performed de-crosslinking followed by DNA extraction using the PCI method. An optimal crosslinking must allow reversal of the process by heating (Das et al. 2004) and should result in a maximum recovery of DNA by the PCI extraction (Haring et al. 2007, Ricardi et al. 2010). We conclude that the crosslinking duration should be limited to a maximum of 30 min and suggest performing crosslinking in batches, 15 min for excising buds from the canes followed by 15 min of crosslinking.

#### Chromatin extraction

In lignified tissues, the presence and composition of secondary metabolites creates a requirement to optimize extraction conditions, particularly the composition of the homogenization buffer and presence and concentration of detergent used for cell lysis (Li et al. 2014*b*). A powerful homogenizer such as the ULTRA-TURRAX (IKA, Germany) is also strongly recommended to improve tissue homogenization. Moreover, polyvinylpyrrolidone (PVP) has been used routinely in nuclei acid extraction from tissue with high polyphenol content (Lodhi et al. 1994, Porebski et al. 1997). Secondary metabolites, such as polyphenols and tannins, can bind to DNA upon cell lysis and contaminated DNA may present problem for downstream analysis, such as DNA library construction for sequencing. The PVP binds polyphenols through hydrogen bonding and can then be removed from tissue homogenate by discarding the supernatant containing PVP-polyphenols after centrifugation step (John 1992). There are also several considerations in the choice and amount of detergent. Typically, an anionic detergent such as sodium docecyl sulfate (SDS) is used, however while concentrations >0.1% SDS (w/v) will improve nuclear isolation, this may disrupt the antibody–antigen interaction due to protein denaturation (Privé 2007). Moreover, high concentrations of ionic detergent tend to result in formation of precipitates at low temperature, risking inefficient cell lysis and co-precipitation with the DNA (Linke 2009). Two concentrations of SDS commonly used in ChIP assays were tested here, 0.1% and 1%, to determine the optimum condition resulting in the highest yield of DNA for immunoprecipitation. Also, we tested 0.1% sarkosyl, a milder anionic detergent, which is structurally similar to SDS but remains soluble under low temperature, as a comparison to the widely use SDS (Linke 2009). Our result show that lower detergent concentration, both ionic and anionic, resulting a low DNA yield (Figure 5, lanes 1, 3 and 5). However, the result was improved after sonication was applied for several minutes.

#### DNA fragmentation

The most common procedures to shear DNA for ChIP assay is by sonication (Orlando et al. 1997, Orlando 2000) or micrococcal nuclease treatment (O’Neill and Turner 2003); the former method is mainly used for crosslinked ChIP experiment. Ideally, DNA is sheared into small fragment range from 200 to 600 bp (Park 2009). Sonication is highly variable and difficult to optimize. A titration approach is commonly required to find the best sonication duration and settings. By considering this, we then performed a test to determine the sonication duration that will produce the desired fragment size. Here, we use S220 Focused-Ultrasonicator (Covaris, USA) and followed manufacture recommendation to generate homogenously distributed ~300 bp DNA fragment, 5% Duty Cycle, 4 intensity, 140 W peak incident power, 200 cycles per burst. We then tested three sonication durations, 6, 8 and 10 min. Fragmented DNA was then analyzed using TapeStation^®^ (Agilent, Australia) and quantified using Qubit (Thermo Fischer Scientific, Australia) as both methods provide a more sensitive and accurate measurement comparatively to measurement using agarose gel or nanodrop respectively (Simbolo et al. 2013). The sonication step served two purposes in our protocol, improve cell lysis and DNA fragmentation. Aggregated nuclei are a common problem when isolating nuclei from tissue with high tannic acid content (Loureiro et al. 2006) and clumping nuclei will also reduce the efficiency of DNA fragmentation (Arrigoni et al. 2015). The development of a standard ChIP protocol using animal cells also demonstrates that mild sonication can help to separate clumping cells which then improves cell lysis process and increase DNA yield (Arrigoni et al. 2015). In agreement with this report, our result showed that the use of high detergent concentration for cell lysis could be avoided using our sonication settings. In addition to improve cell lysis, our sonication setting was found to be affected long DNA more than short DNA. Library construction may increase bias toward short DNA fragments due to size selection during library construction. Although 10 min sonication was sufficient to shear grapevine chromatin into a suitable size for sequencing (usually within 150–300 bp range), we suggest applying 12 min of sonication in order to obtain a higher amount of DNA fragment within the 150–300 bp range.

#### Antibody validation

A specific antibody with a high affinity to the protein of interest is a prerequisite for a successful ChIP experiment (Kungulovski et al. 2015). Antibodies are common tools for studying many biological processes; however, they may also cause problems (Saper and Sawchenko 2003, Baker et al. 2015). Common problems are (i) recognition of non-target protein due to antibody cross-reactivity, (ii) non-reproducible results due to batch-to-batch variation of antibody and (iii) unsuitable application, for example antibodies that work for western blotting may not be suitable for immunoprecipitation (Baker et al. 2015). It is imperative to characterize and validate the antibody of choice before commencing an experiment (Schumacher and Seitz 2016, Gautron 2019). Egelhofer et al. (2011) tested 246 ChIP-grade antibodies and found that any of these antibodies were either non-specific or unsuitable for ChIP. In order to address this issue, we performed antibody assessment to validate the ChIP antibody that was used in our experiment. We chose antibodies for histone H3, H3K4me3 and H3K27me3 on the basis of existing public data on the specificity, in order to meet at least one of the selection criteria. The antibodies chosen had been shown to specifically recognize the antigen in HeLa cells by the manufacture, in various human or mouse tissue by the ENCODE project and used in ChIP analysis in barley (Baker et al. 2015). Recombinant histone H3 and nuclear extract of grapevine buds were tested against anti-histone H3, anti-H3K4me3 and anti-H3K27me3. Criteria for an antibody to ‘pass’ specificity by western blotting was adopted from Egelhofer et al. (2011), the tested antibody should produce at least 50% signal compare to the total nuclear signal and ten-times higher than any unspecific signal. As such, the anti-histone H3 (unmodified) and anti-H3K4me3 passed specificity however the anti-H3K27me3 was inconclusive. In future studies it would be appropriate to test alternative batches or suppliers for the modified antibodies, however, this is not always practical, as was the case in the present study.

### ChIP-sequencing and histone H3 occupancy

The outcome of the ChIP experiment is fragments of DNA that specifically interact with the protein of interest. Identification of the DNA sequence following the immunoprecipitation can be done by polymerase chain reaction (ChIP-PCR) or quantitative PCR (ChIP-qPCR), microarray (ChIP-chip), and high-throughput sequencing (ChIP-seq). Endpoint PCR or qPCR is the most widely and routine identification technique use in ChIP. The pitfall of this technique is that it requires prior knowledge of regions associated with the protein tested. Rapid improvement of genome-wide assays using microarray or high-throughput sequencing, provides an alternative DNA assay for species such as grapevine; in which knowledge about the region occupied by histone H3 or modified histone H3 is not available. Several reviews outline the superiority of sequencing over microarray for several reasons, such as higher genome coverage including the repeated sequence and low noise to signal ratio which is commonly found in microarray analysis (Schones and Zhao 2008, Park 2009, Furey 2012). In this study, we performed ChIP-seq analysis of the histone H3 to evaluate our ChIP protocol. We also compare and explore the histone H3 occupancy along grapevine bud chromatin using dormant buds harvested at three different time point.

The study of histone H3 occupancy during embryo development or bud dormancy is still very limited compared to nucleosome (histone octamer) occupancy. Nucleosome (histone octamer) occupancy and positioning have been suggested to play important roles in regulating gene expression and many additional DNA-related processes (Struhl and Segal 2013). Studies of nucleosome occupancy and positioning in animals, yeast and plant cells have demonstrated a bias in nucleosome occupancy positioning toward regions proximal to the TSS (Mavrich et al. 2008, Schones and Zhao 2008, Zhang et al. 2015, Lee et al. 2017). Furthermore, genome-wide nucleosome occupancy studies in yeast, mammalian and plant systems show that the genomic sequence of nucleosome is mostly depleted in the promoter or transcription termination sites (Field et al. 2008, Fenouil et al. 2012, Liu et al. 2015). In yeast, nucleosome depletion was found in the homopolymers of deoxyadenosine nucleotides (poly (dA:dT) tracts) regions, suggesting that the structure of poly (dA:dT) tracts may be resistant to the bending and twisting deformation required to wrap DNA around nucleosomes (Field et al. 2008, Segal and Widom 2009 and the reference therein). On the contrary, in mammalian and plant tissues, promoter regions are mostly GC-rich, hence the nucleosome depletion is tightly associated with CpG islands (Fenouil et al. 2012, Liu et al. 2015). When compared to these studies in nucleosome occupancy, we observed similar pattern of histone H3 occupancy, higher preference occupation at downstream TSS region and genic region. Functional analysis of genes associated with histone H3 at the genic region showed enrichment of process regulating bud growth at multiple levels, the nucleotide metabolism and embryo morphogenesis in March, cell growth and transition from vegetative to reproductive phase in May, and response to cold in August. A study observing transcriptome variation during grapevine bud development suggested that upregulation of process related to nucleotide metabolism in endodormant buds may be related to accumulation of translatable mRNA as was previously reported in dry *Arabidopsis* seeds while cell growth was enriched in transition from ecodormant to budbreak (Kimura and Nambara 2010, Díaz-Riquelme et al. 2012). In addition, similar categories related to process preceding the endodormancy release were observed in buds harvested in May and treated with H_2_CN_2_, this includes genes associated reactive oxygen species detoxification, sugar signaling and ABA-mediated signaling (Díaz-Riquelme et al. 2012, Ophir et al. 2009, Halaly et al. 2008).

Differential expression and abundance of histone H3 during embryo development in animals and plants were reported to correlate well with DNA synthesis and cell-cycle activities, showing the highest abundance during early embryogenesis in *Drosophilla* (Shindo and Amodeo 2019), or in cycling cells of plant meristems (Kapros et al. 1992, Terada et al. 1993, Sano and Tanaka 2005) and at low abundance in quiescent apical buds (Singh et al. 2009). These studies indicate that actively dividing cells maintain a high amount of histone H3 and reduce it when cell division is limited. A recent report on the grape bud dormancy in the Western Australia region (southern hemisphere), showed a non-traditional dormancy characteristic that dormancy peak (increase in BB_50_) in summer (March), followed by a rapid increase in the capacity to resume growth (decline in BB_50_) at the beginning of winter (May), and maintaining quiescent state until spring (Velappan et al. 2022). Comparing the bud burst profile with the overall histone H3 occupancy in this study, we found that the number of genic regions associated with the histone H3 were found to be the highest when buds are at the stage of release from dormancy, in May (5 W) and when treated with H_2_CN_2_ (3H). While the lowest, was observed when buds were in the dormant (March/3 W) and quiescent (August/8 W) stages. It may be indicated here that when the bud resumed its capacity to grow in May or due to H_2_CN_2_ application, the cells prepared for cell cycle resumption and begun to actively express histone H3 protein. However, the external temperature from May to August was still unfavorable for bud burst; therefore, the cells delayed the progression of cell cycle, histone H3 protein was not maintained further, and the abundance was found to decrease in August. Although more replicates are needed so that it is statistically sufficient to draw a conclusion, our results showed there is a corresponding trend between histone H3 abundance with grape bud dormancy state.

## Conclusion

We describe the systematic optimization of detail chromatin immunoprecipitation protocol for grapevine bud samples. The protocol was developed from chromatin immunoprecipitation (ChIP) protocol for woody tissue published by Li et al. (2014*b*) and then modified according to optimization results that we performed at each step of the ChIP protocol; this included the amount of starting material, crosslinking method, chromatin extraction condition, chromatin shearing duration, validation of antibody and DNA purification method. Identification of histone H3 enriched DNA by sequencing, provided an example for the potential use of this protocol to study the post-translational modification of histone H3 in the buds of grapevine.

## Supplementary Material

SuppTable1_qPCR_titration_tpac146

SuppTable2_Sequencing_Statistics_tpac146

SuppTable3_Annotated_peaks_histoneH3_Chip-Seq_tpac146

SuppTable4_GO_enrichment_genic_region_tpac146

SuppTable5_5W3H-Intersection_tpac146

SuppMethS1-S4_tpac146

## Data and materials availability

All datasets generated for this study are included in the article/supplementary material.
