# Supplementary material for: Systematic evaluation of chromatin immunoprecipitation sequencing to study histone occupancy in dormancy transitions of grapevine buds
Source: Tree Physiol. 2023 Jan 13;43(4):675–89. doi: 10.1093/treephys/tpac146 (PMC10094961; doi:10.1093/treephys/tpac146)
Supplement: SuppMethS1-S4_tpac146 [file suppmeths1-s4_tpac146.docx]

## Supplementary Information

## S.1 Chromatin Immunoprecipitation Procedure

### Tissue collection

1. The buds from node 4 to 7 were excised from the cane and dissected into half, longitudinally, to increase surface exposed to crosslinking buffer then immediately immersed in a fixative solution. We used whole buds in this experiment for the convenience of bud harvesting.

### Crosslinking

1. Immediately put the bud into conical tube contains 25 mL **CROSSLINKING BUFFER**, repeat this until 100 buds are obtained (ca. 2.5 grams). Crosslink the buds for a total of 15 minutes under cycled vacuum infiltration (5 min/ release/ mix, repeat three times) at room temperature.

**NOTE**: Excessive exposure to crosslinking agents may result in inefficient DNA fragmentation and protein denaturation. Since buds need to be excised from the canes for this experiment, it took some time to harvest 5-10 grams of buds. We suggest cutting as many buds as possible in 30 minutes then immediately proceed with vacuum infiltration. In our case, we handled 100 buds at a time.

1. Quench the crosslinking reaction by addition of 2 M glycine to a final concentration of 200 mM, followed by 5 minutes cycled vacuum.
2. Rinse crosslinked tissue with water twice. Dry tissue between absorbent paper then put them on the foil.
3. Snap freeze tissue in liquid nitrogen and store at -80 °C until required.

### Nuclear isolation

1. Unless otherwise indicated, all step must be performed at 4 °C, and the sample must be kept on ice all the time.
2. Grind crosslinked buds to a fine powder in liquid nitrogen using mortar and pestle. Always grind a small amount of tissue at a time, then collect powder into a new 50 mL conical tube. Repeat grinding until all 10 grams of crosslinked buds are ground. The conical tube must be kept on dry-ice all the time.

**NOTE**: one 50 mL canonical tube is suitable for 5 grams of tissue powder. When working with 10 grams tissue, split the ground powder into two new tubes.

1. Mix the powder with seven volumes of **BUFFER 1** in 50 mL conical tube (e.g. 35 mL for every 5 grams tissue). Homogenize using a vortex and an ULTRA-TURRAX homogenizer at 9000 rpm for 15 seconds. Further mix suspension in rotating wheel for 20 minutes at 4 °C.

**NOTE**: Complete homogenisation is important to get a maximum DNA yield.

**CHECKPOINT**: Comparing DNA yield obtained from vortex homogenization vs ULTRA-TURRAX may be needed to optimise the homogenisation method.

1. Pass the mixture through three layers of Miracloth saturated with Buffer 1 into new 50 mL conical tube. Squeeze the Miracloth to collect all the liquid.
2. Centrifuge suspension at 2,880 *g* for 10 minutes at 4 °C. Discard supernatant.
3. Gently resuspend pellet in 2 mL of **BUFFER 2** and transfer suspension into a new 2 mL microcentrifuge tube.
4. Centrifuge suspension at 12,000 *g* for 10 minutes at 4 °C. Discard supernatant.
5. Repeat step 10 to 12 once.
6. Gently resuspend pellet in 500 µL of **BUFFER 3**. Carefully layer the suspension on top of 1.5 mL cushion of **BUFFER 3** in a new 2 mL microcentrifuge tube.

**NOTE**: Pellet may be difficult to resuspend. A disposable tissue grinder pestle can be used to carefully loosen the pellet followed by pipetting up and down.

1. Centrifuge sample at 16,000 *g* for 60 minutes at 4 °C. Discard supernatant.
2. Gently resuspend pellet in 700 µL of **LYSIS BUFFER**. Take 50 µL for the no-sonication control and keep the resuspended pellet on ice.

**CHECKPOINT**: check yield of DNA and validate antibody (**Supplementary Methods S3** and **S4**).

**CHECKPOINT**: Nuclei integrity can be checked by adding DAPI dye to a final concentration of 10 mg/mL and examine nuclei using an epiluminescence microscope (**Figure 6**).

### DNA fragmentation

1. Transfer nuclei suspension into miliTUBE being sure to fill the tubes with lysis buffer (a little more than 1 mL per tube).
2. Sonicate the DNA in Covaris S220 focus-ultrasonicator for 12 minutes following manufacture’s setting for high cell chromatin shearing, i.e. 5 % Duty Cycle, 4 intensity, 140 W peak incident power, 200 cycles per burst, 6 °C bath temperature, frequency sweeping power mode, continuous degassing mode, and level 8 water. Transfer sonicated DNA into a new 1.5 mL.

**CHECKPOINT**: Take 50 µL aliquots after 6, 8, and 10 minutes to compare DNA fragmentation and each time replace with the same amount of lysis buffer. Keep sample on ice.

1. Centrifuge sonicated and non-sonicated DNA at 16,000 *g* for 10 minutes at 4 °C. Transfer clean supernatant into a new 1.5 mL microcentrifuge tube.
2. Proceed immediately to step 21 for chromatin immunoprecipitation. DNA can be stored at – 20 °C and proceed to **Supplementary Methods S3** for DNA fragmentation efficiency examination.

### Chromatin immunoprecipitation and reverse crosslinking

The following chromatin immunoprecipitation and reverse crosslinking procedure are adapted from ChIP kit plant from Abcam with some modification.

1. Determine the number of strip wells required. Leave these strips in the plate frame (remaining unused strips can be placed back in the bag. Seal the bag tightly and store at 4 °C).
2. Wash strip wells once with **150 μL** of **WASH BUFFER**.
3. Add 100 μL of the **ANTIBODY BUFFER** to each well and then add the antibodies:

- **3 μg** of an antibody of interest (H3K27me3 and H3K4me3).
- **2 μg** of anti-histone H3 as a positive control.

**NOTE**: ChIP typically requires 1-10 µg per ChIP reaction. Optimising the amount used per reaction is a further variable to consider, however here the amount chosen followed manufacturer recommendations.

In our experiment with grapevine buds, three reactions (wells) were prepared for each histone H3 modified antibody and two reactions for histone H3 antibody (**Figure S1**).


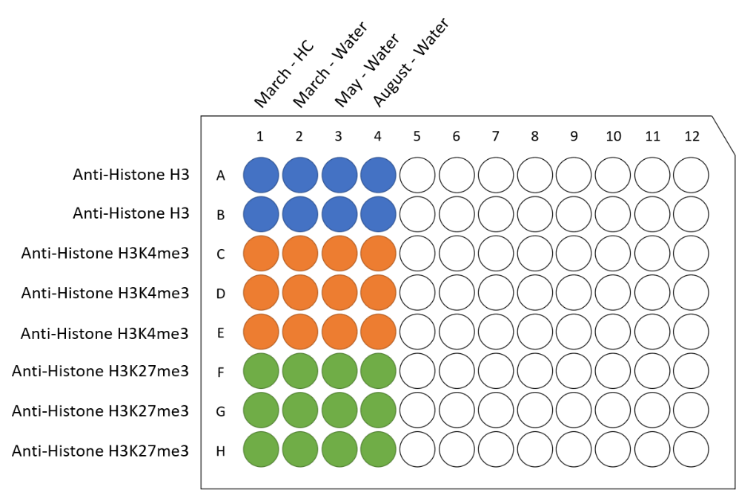


**Figure S1**. ChIP assay plate map. Incubation of chromatin and antibodies is done in an assay-well provide in Abcam’s ChIP kit plant. Each well is designed for one ChIP reaction using 100 µL fragmented DNA. In our experiment, multiple wells were used per antibodies, i.e. 2-well for anti-histone H3 (blue) and 3-well each for anti-Histone H3K4me3 (orange) and anti-Histon H3K27me3 (green), with each column represent different sample.

1. Cover the strip wells with **Parafilm M** and incubate at room temperature for 90 minutes.
2. After incubation, remove the incubated antibody solution and wash the strip wells **three times** with **150 μL** of the **ANTIBODY BUFFER** by pipetting in and out.
3. Remove **15 μL** of chromatin aside to a 0.5 mL vial. Label the vial as **“input DNA”** and then place on ice.

**NOTE**: the amount of input DNA is 5 % from the total volume of chromatin used per histone H3 modifies antibodies, i.e. 5 % from 300 µL.

1. Transfer **100 μL** of **chromatin from step 19** to each antibody-bound strip well. Two and three reactions (wells) are used for Histone H3 and Histone H3-modified immunoprecipitation.

**NOTE**: Concentration of SDS in LYSIS BUFFER (step 15) is 0.1 %; therefore, no sample dilution needed.

1. Cover the strip wells with **Parafilm M** and incubate at 4 °C for overnight on an orbital shaker (50-100 rpm).
2. Remove supernatant. Wash the wells six times with **150 μL** of the **WASH BUFFER**. Allow 2 minutes on a rocking platform (100 rpm) for each wash.
3. Wash the wells once (for 2 minutes) with **150 μL of 1X TElowE BUFFER**.
4. Add **40 μL of the DNA Release mix**, containing 1 µL Proteinase K (10 mg/mL) and 40 µL DNA RELEASE BUFFER, to the samples (including the “input DNA” vial).
5. Cover the sample wells with strip caps and incubate at 65 °C in a water bath for 15 minutes. Following incubation at 65 °C do a quick spin to collect all suspension at the bottom of the plate.
6. Add **40 μL of the REVERSE BUFFER** to the samples and to a vial labelled as “input DNA”; mix and re-cover the wells with strip caps and incubate at 65 °C in a water bath for 90 minutes. Quick spin plate at RT.
7. Combine solution from the same histone antibody (2 wells for Histone H3 and 3 wells for Histone H3 modified).

### DNA purification with AMPure Beads

1. Add 1.8X volume of AMPure XP beads to IP enriched and input DNA.

**NOTE**: This step will bind DNA fragments size from 100 bp and larger.

1. Mix reagent and sample thoroughly by **pipette mixing** **ten times**.
2. Let mixed samples incubate for 15 minutes at room temperature for maximum recovery.

**NOTE**: pipette mixing is preferable to vortexing as it tends to be more reproducible. The colour of the mixture should appear homogenous after mixing.

1. Place on a magnetic rack for 5 minutes (wait for solution to clear before proceeding to the next step).
2. With tube still in the magnetic rack, aspirate the clear solution from tube and discard.
3. Keep the sample in magnetic rack and add 1 mL of freshly prepared 70 % ethanol.
4. Incubate for 30 seconds at room temperature. Aspirate out the ethanol and discard.
5. Repeat ethanol wash one more time.
6. Illumina recommended at least 15 minutes drying time but longer drying time may be required.

**NOTE**: ensure all traces of ethanol are removed but avoid over-drying the beads, which will significantly decrease elution efficiency (beads will appear cracked if over dried).

1. Remove the tube from the magnetic rack, add 10 μL TElowE and pipet up and down several times until pellet beads are completely resuspended.

**NOTE**: Standard TE **must not be used** at this step.

1. Incubate at room temperature for 2 minutes. Place in the magnetic rack for 5 minutes.
2. Transfer 9 μL of the supernatant to a 0.2 mL PCR tube.
3. Repeat step 44-46 once. DNA is now ready for use or store at – 20 °C.

## S.2 SimpleChIP® ChIP-seq DNA Library Prep Kit for Illumina® Protocol

### End preparation

Before starting prepare 5 ng enriched-DNA and thaw End Prep Reaction Buffer (green cap) at room temperature.

1. Prepare DNA by adding 1× lowE-TE to generate a final volume of 50 μl for each DNA sample.
2. Add 10 μl End Prep master mix (below) to each DNA sample.

| **Component** | **Volume/ rxn** |
| --- | --- |
| End Prep Enzyme mix | 3 µL × |
| End Prep Reaction Buffer | 7 µL × |

1. Thoroughly mix the reactions by pipetting up and down at least 10 times and perform a quick spin to collect all liquid from the sides of the tube.
2. Place in a thermocycler and run with the following program: 30 minutes at 20 °C, 30 minutes at 65 °C, and hold at 4 °C. Make sure the lid heat is on at 75 °C.
3. Proceed to Adaptor Ligation.

### Adaptor ligation

Before starting, thaw Adaptor for Illumina at room temperature, mix the Ligation Master Mix by pipetting up and down several times.

1. Make a 1:10 dilution (1.5 μM working concentration) of the Adaptor in 10 mM Tris-HCl (pH 8.0-8.5)
2. Prepare Adaptor ligation Master mix as the following:

| **Component** | **Volume/rxn** |
| --- | --- |
| Ligation master mix | 30 µL × |
| Ligation enhancer | 1 µL × |

**The Ligation Master Mix and Ligation Enhancer can be mixed ahead of time and is stable for at least 8 hours @ 4 °C. Addition of adaptor into Adaptor Ligation mix is not recommended.**

1. Add 2.5 μl diluted Adaptor directly to the 60 μl End Prep Reaction Mixture from step.
2. Add 31 µL Adaptor ligation master mix.
3. Thoroughly mix the ligation reaction by pipetting up and down at least 10 times and perform a quick spin to collect all liquid from the sides of the tube.
4. Place mixture in thermo cycler and incubate at 20 °C for 15 minutes. Make sure lid heat is OFF.
5. Add 3 μl of USER Enzyme (red cap) to the ligation mixture, mix well and placed back into thermal cycle for incubation at 37 °C for 15 minutes. Set lid heat to 47 °C.
6. Proceed to Cleanup of Adaptor-ligated ChIP DNA.

**SAFE STOP**: At this point, samples can be stored overnight at -20 °C.

### Cleanup of adaptor-ligated ChIP DNA without size selection

Size selection is NOT recommended during the Cleanup Adaptor-ligated ChIP DNA phase, because it results in a dramatic decrease in both yield and diversity of ChIP-seq DNA libraries.

Before starting allow the AMPure® XP beads to warm to room temperature for at least 30 minutes before use and xortex the beads to resuspend. Prepare 400 μl of 80% ethanol for each sample.

1. Add 96.5 μl (1X) resuspended AMPure XP beads to each adaptor ligation reaction. Mix well by pipetting up and down at least 10 times. Incubate samples on bench top for at least 5 minutes at room temperature.
2. Place the tube/plate on an appropriate magnetic stand for 5 minutes to separate the beads from the supernatant. Once the solution is clear, carefully remove and discard the supernatant. Be careful not to disturb the beads that contain DNA targets.
3. Add 300 μl of 80% freshly prepared ethanol to the tube/plate while in the magnetic stand. Incubate at room temperature for 30 seconds, and then carefully remove and discard the supernatant. Be careful not to disturb the beads that contain DNA targets.
4. Repeat ethanol wash (step 16).
5. Air dry the beads for up to 5 minutes while the tube/plate is on the magnetic stand with the lid open.
6. Remove the tube/plate from the magnetic stand. Elute the DNA target from the beads by adding 17 μl of 10 mM Tris-HCl (pH 8.0-8.5) per sample.
7. Mix well by pipetting up and down 10 times. Incubate for at least 5 minutes at room temperature.
8. Place the tube/plate on the magnetic stand and wait for 5 minutes. Once the solution is clear, transfer 15 μl of supernatant containing the DNA targets to a new PCR tube.
9. Proceed to PCR Enrichment of Adaptor-ligated ChIP-DNA.

**SAFE STOP**: Alternatively, samples can be stored at -20 °C.

### PCR enrichment of adaptor-ligated ChIP DNA

Before starting: Thaw primers and the purified adaptor-ligated ChIP DNA fragments (from step 21 if sample is stored at -20 °C) at room temperature.

1. Add the following components to a sterile PCR tube:

| **Reagents** | **Volume/ rxn** |
| --- | --- |
| Purified adaptor ligated ChIP-DNA fragments (from step 21) | 15 µL × |
| Q5® PCR Master Mix | 25 µL × |
| Dual Index 7 Primer for Illumina | 5 µL × |
| Dual Index 5 Primer for Illumina | 5 µL × |

1. Thoroughly mix the reaction by pipetting up and down 10 times and perform a quick spin to collect all liquid from the sides of the tube.
2. Place the tube on a thermo cycler and perform PCR amplification using the following PCR cycling conditions:

| a. | Initial denaturation | 98°C for 30 sec |
| --- | --- | --- |
| b. | Denaturation | 98°C for 10 sec |
| c. | Anneal and extension | 65°C for 75 sec |
| d. | Repeat steps b and c for a total of  10 cycles, for 2.5 < ChIP DNA ≤ 5 ng  11 cycles, for 1.25 ng < ChIP DNA ≤ 2.5 ng,  12 cycles, for ChIP DNA ≤ 1.25 ng | |
| e. | Final extension | 65°C for 5 min |
| f. | Hold | 4°C |

1. Proceed to Cleanup of PCR Amplification.

**SAFE STOP**: Alternatively, samples can be stored at -20 °C.

### Cleanup of PCR amplification

Before starting, allow the AMPure® XP beads to warm to room temperature for at least 30 minutes before use and xortex the beads to resuspend. Prepare 400 μl of 80% ethanol for each sample. Prepare approximately 40 μl of 10 mM Tris-HCl (pH 8.0-8.5) for each sample.

1. Add 45 μl (0.9X) resuspended AMPure XP beads to 50 μl PCR reaction from step 26. Mix well by pipetting up and down at least 10 times. Incubate samples on bench top for at least 5 minutes at room temperature.
2. Place the tube/plate on an appropriate magnetic stand for 5 minutes to separate the beads from the supernatant. Carefully remove and discard the supernatant. Be careful not to disturb the beads that contain DNA targets.
3. Add 200 μl of 80% freshly prepared ethanol to the tube/plate while in the magnetic stand. Incubate at room temperature for 30 seconds, and then carefully remove and discard the supernatant.
4. Repeat ethanol wash (step 29). Be sure to remove all visible liquid after the second wash.
5. Air dry the beads for up to 5 minutes while the tube/plate is on the magnetic stand with the lid open.
6. Remove the tube/plate from the magnetic stand. Elute the DNA target from the beads by adding 33 μl of 10 mM Tris-HCl (pH 8.0-8.5) per sample. Mix well by pipetting up and down 10 times. Incubate for at least 5 minutes at room temperature.
7. Place the tube/plate on the magnetic stand and wait for 5 minutes. Carefully transfer 30 μl of supernatant containing the DNA targets to a new tube.

**SAFE STOP**: Libraries can be stored at -20°C.

1. Measure the concentration of library DNA using Qubit fluorometer. The concentration of the DNA library should be in the range of 10-40 ng/μl.
2. Dilute 1 μl of the library DNA with 10 mM Tris-HCl to a final concentration of 5-10 ng/ μl and use the diluted library DNA to determine size distribution using bioanalyzer.

**NOTE**: If adaptor dimer (~128 bp for Single Index Primers or ~146 bp for Dual Index Primers contamination is observed, repeat clean up (step 27-33). Residual adaptors and/or adaptor dimers will strongly contaminate the sequencing reaction.

## S.3 DNA analysis

Chromatin reverse crosslinking must always be performed when checking DNA yield and crosslinking and DNA fragmentation efficiency. DNA purification can be done using phenol/chloroform/isolamyl alcohol (PCI), Solid Phase Reversible Immobilisation (SPRI) paramagnetic-based beads, or column method. We recommend using SPRI beads method for ChIP-enriched DNA purification, however PCI method can be used for CHECKPOINT purposes.

### De-crosslinking

1. Adjust volume of the aliquot from step 15 or 19 to 500 µL using TElowE buffer (pH 8).
2. Add 5 M NaCl to clear supernatant of chromatin with final concentration of 0.2 M (e.g to 500 µL chromatin add 20 µL 5 M NaCl).
3. Mix well and incubate at 65 °C for 4 hours or over the night.
4. Add proteinase K (20 mg/mL) to final concentration of 14 mg/mL and RNase A (10 mg/mL) to final concentration of 0.31 µg/µL, then incubate at 45 °C for an hour.
5. Proceed to DNA purification. For PCI method follow step 53 to 58 and for SPRI beads method follow step 35-47.

### Phenol/Chloroform/ Isoamyl alcohol DNA Purification

1. Add equal volume of Phenol:Chloroform:Isoamyl alcohol (25:24:1) and mix by gently inversion several times.
2. Centrifuge at 12,000 *g* for 10 minutes and transfer upper aqueous phase into new 1.5 mL microcentrifuge tube.
3. Add 2.5 volume of absolute ethanol and 0.1 volume of 3 M sodium acetate (pH 5.2), mix gently by pipetting up and down. Incubate on ice for an hour followed by at – 20 °C for over the night.
4. Centrifuge at 16,000 g for 30 min at 4 °C. Discard supernatant.
5. Wash pellet with 500 µL 70 % ethanol, centrifuge again at 16,000 *g* for 30 in at 4 °C. Decant supernatant and air-dry pellet.
6. Dissolve pellet in 20 µL TElowE buffer.
7. Check quantity and quality of the DNA using 1.5 % agarose gel or TapeStation and nanodrop or Qubit fluorometer.

## S.4 Antibodies Validation

Performance of ChIP-grade antibody is examined against total isolated chromatin and recombinant Histone H3 protein. Reverse crosslinking of total chromatin is not required at this step.

### Protein Gel Electrophoresis

1. Add 1X **LAEMMLI SAMPLE BUFFER** into nuclear extract and denature in boiling water for 3 minutes.

**NOTE**: loading too much protein can result in unspecific antibody reaction in immunoblot, therefore concentration of nuclear extract and recombinant protein must be pre-determined prior to assessment. We recommend referring to Egelhofer et al. (2011) as guidelines to decide the most suitable concentration.

**NOTE:** this sample can be store at – 20 °C for later usage. The rest of protein stock (indentured) can be stored in – 80 °C for a long period storage.

1. Load 20 µL of the denatured sample and protein ladder onto an SDS-polyacrylamide gel.

**NOTE**: Typically, electrophoresis is carried out at a constant current of 25 mA for a small gradient gel. Electrophoresis is usually performed until the bromophenol blue dye has run off the bottom of the gel or approximately an houe. Because the dye front also contains the free labelled amino acids, disposal of unincorporated label may be easier if the gel is stopped while the dye front remains in the gel.

1. Immediately transfer gels in casts to ChemiDoc-MP to visualize equal loading by TGX stain-free method. Remove gels from casts and capture the image.

**NOTE**: see step 88 to 97 for guideline using ChemiDoc-MP

1. Carefully store gels back into the pre-wetted containers. Proceed to semi-dry blotting.

**Gel Transfer and Semi-Dry Western Blot**

### Membrane & blot papers preparation

1. Prepare transfer buffer by adding 20 % methanol into **RUNNING BUFFER**. Pre-wet and equilibrate PVDF membrane in methanol for 30 seconds, discard methanol and wash twice with **TRANSFER BUFFER** @ 5 minutes.
2. Then leave equilibrated PVDF membrane in transfer buffer on a rocking platform.
3. Pre-wet and equilibrate blot papers (4 per sandwich) with **TRANSFER BUFFER** and leave them on a rocking platform.

### Gel transfer and blotting

1. Equilibrate the gel in a small container of transfer buffer for 10 minutes.
2. Place 2 pieces of pre-soaked blot paper onto the platinum anode. Remove bubbles carefully by using a roller.
3. Place the pre-wetted and equilibrated PVDF membrane on top of the wetted blot paper.
4. Carefully place the equilibrated gel on top of the PVDF membrane. Avoid bubbles or air pockets, and use roller if bubble is formed.
5. Place 2 piece of pre-soaked blot paper on top of the gel. Remove bubbles.
6. Add a bit more of the transfer buffer to the sandwich to help avoiding a short circuit error when running or sop up transfer buffer with paper towel if too wet.
7. Place the cathode plate onto the stack. Press to engage the latches with the guideposts without disturbing the gel/membrane stack.
8. Run the transfer unit at 15 V for 30 minutes or 35 mA for 1-1.5 hours.
9. Retain the membrane and wash 2X with DI water for each 5 minutes.
10. Wash again with TBST for 5 minutes.

### Blocking the membrane

1. Incubate the membrane in blocking buffer at 4 °C for over the night, with gentle agitation on rocking platform. Then pour off the **BLOCKING BUFFER**.
2. Wash 2X with **TBST** @10 mins.

### Primary Antibody Binding

1. Prepare 10 mL of a 1:1,000 primary antibody in **BLOCKING SOLUTION** (that is adding 10 μL into 10 mL blocking solution).
2. Incubate the membrane in the diluted primary antibody for over the night with gentle shaking at 4 °C.
3. Wash the membrane by incubation in 15 mL of **TBST** for 10 minutes with gentle shaking. Repeat the wash two more times using **TBST**.

### Secondary Antibody Binding (HRP-conjugated antibody)

1. Prepare 10 mL of a 1:10,000 secondary antibody in **BLOCKING SOLUTION** (that is adding 1 μL into 10 μL blocking solution).
2. Incubate the membrane in the diluted secondary antibody for 2 hours with gentle shaking.
3. Wash the membrane by incubation in 15 mL of **TBST** for 5 minutes with gentle shaking. Repeat the wash two more times using **TBST**, then three times using 15 mL DI water.

### Preparation of Substrate

1. Mix detection reagents 1 and 2 at a 1:1 ratio and add it to the blot. Incubate blot for a minute.
2. Drain excess reagent. Cover blot with a clear plastic sheet protector or clear plastic wrap.

### Chemiluminescence detection

1. Turn on the Chemidoc MP imaging system. Click on *Image Lab 5.2* icon.
2. Select acquisition setting to Gel imaging. In the application menu, set to Blots and Stain Free Gel. Select Mini PROTEAN for imaging area.
3. In the Image exposure menu, set the Signal Accumulation Mode. **Figure 10** show an example for Signal Accumulation Mode.
4. If the total number of image is set to more than 1, the instrument will be showing several immunoblot images. Choose for the best image and save image by go to file 🡪 export 🡪 export for publication 🡪 select designated folder.


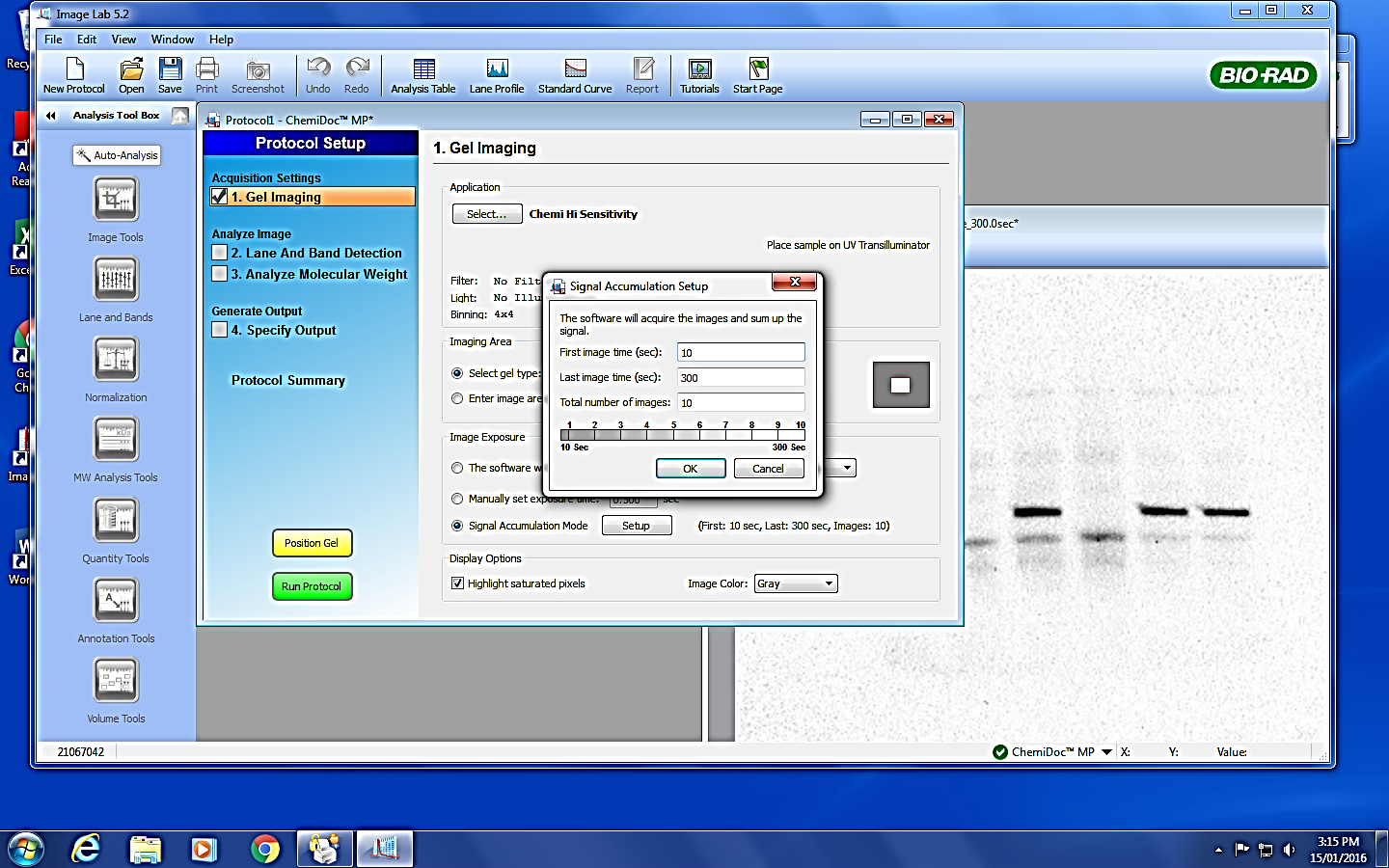


**Figure S4.1** Signal Accumulation Mode setup in the Chemidoc MP software.

1. For colorimetric images, select Blots 🡪 colorimetric in the Application menu. To merge colorimetric and gel images, go to image tools and click on merge.
2. For gel imaging before protein transfer to blot, set Application to Protein Gels then select Stain free gels. Set gel activation to 1 minute, or 2.5 minutes for good sensitivity, or 5 minutes for a maximum sensitivity.

## S.5 Reagents, equipment, and reagent setup

### Reagents

- Sucrose (Chem-Supply, Australia, cat. no. SA030-500G)
- UltraPure 1 M Tris-Cl pH8 (Invitrogen, Australia, cat. no. 15568-025)
- 0.5 M EDTA pH 8 (Invitrogen, cat. no. AM9260G)
- Paraformaldehyde (Sigma-Aldrich, Australia, cat. no. P6148-500G)
- Glycine (Chem-Supply, cat. no. GA007-500G)
- β-mercaptoethanol (Sigma-Aldrich, cat. no. 63689-100ML-F)
- Polyvinylpyrrolidone (Sigma-Aldrich, cat. no. PVP40-100G)
- Triton X-100 (Sigma-Aldrich, cat. no. T9284-100ML)
- NaCl (Sigma-Aldrich, cat. no. S7653-1KG)
- Sodium dodecyl sulfate (SDS, Merck, Australia, cat. no. 8.17034.1000)
- Hydrochloric acid (Sigma-Aldrich, cat. no. 320331-2.5L)
- Miracloth (Merck-Millipore, Australia, cat. no. 475855)
- UltraPure phenol:chloroform:isoamyl alcohol 25:24:1 (v/v) (Invitrogen, cat. no. 15593031)
- Ethidium bromide (Sigma-Aldrich, cat. no. E1510)
- Absolute ethanol (Merck, cat. no. 1.00983.2511)
- Agarose (Thermo Scientific, Australia, cat. no. 16500100)
- 1 kb DNA ladder (Promega, USA, cat. no. G5711)
- Mini-PROTEAN TGX (Tris-Glycine eXtended), 4-15% precast gradient polyacrylamide gel (Biorad, Australia, cat. no. 161-1104EDU, 10-well, 30 µl, 8 x 10 cm (W x L))
- 10x Tris/Glycine/SDS Buffer (Biorad, cat. no. 161-0732)
- 4X Laemmli buffer (Biorad, cat. no. 1610747)
- Protein marker (Blue Star Pre-stained Protein Marker, Nippon Genetics, Japan, cat. no. MWP03)
- Immun-Blot® PVDF membrane, precut, 7 x 8.4 cm (Biorad, cat. no. 1620174)
- Extra thick blot filter paper, precut, 8 X 13.5 cm (Biorad, cat. no. 1703966)
- Trans-Blot® SD Semi-Dry Electrophoretic Transfer Cell (Biorad, cat. no. 1703940)
- Primary antibodies: Histone H3 – nuclear loading control rabbit pAb (Abcam, Australia, cat. no. ab1791), Histone H3K4me3 antibody rabbit pAb (Active Motif cat. no. 39915), Histone H3K27me3 antibody rabbit pAb (Active Motif cat. no. 39155)
- Goat anti-rabbit IgG HRP conjugated secondary antibody (Santa Cruz Biotechnology, cat. no. SCZSC-2030)
- Pierce™ ECL Western Blotting Substrate (Thermo Scientific, Australia, cat. no. 32109)
- ChIP kit plant (Abcam, cat. no. ab117137)
- NEBNext® Ultra™ II DNA Library Prep Kit for Illumina® (New England Biolabs, cat. no. NEB.E7645G).
- NEBNext® Multiplex Oligos for Illumina® (New England Biolabs, cat. no. NEB.E7335G).
- Agentcourt AMPure XP beads (Beckman Coulter Life Science, USA, cat. no. A63881)
- 4',6-Diamidino-2-Phenylindole dihydrochloride (DAPI, Sigma, cat. no. 102M4012V)

### Equipment

- Vacuum chamber
- Vacuum pump
- Aluminium foil
- Conical tubes (50 mL and 15 mL)
- Mortar and pestle
- Rotator
- Vortex (Velp Scientifica, Italy)
- ULTRA-TURRAX homogeniser (model T25 basic, IKA, Germany)
- Refrigerated centrifuge (model 5810R, Eppendorf)
- Fix-angle rotor (model F45-30-11 and F34-6-38, Eppendorf)
- Microcentrifuge tube (1.5 and 2 mL)
- Focus-ultrasonicator (model S220, Covaris, USA)
- miliTUBE 1 mL AFA fibre (Covaris, cat. no. 520130)
- Hot water bath (model B-491, Buchi, Switzerland)
- NanoDrop (model ND-1000, Thermo Fischer Scientific, Australia)
- Qubit fluorometer (model Qubit 3.0, Thermo Fischer Scientific, Australia)
- Bioanalyzer (Agilent 2100 bioanalyzer, Agilent, Australia)
- Electrophoresis system (Mini Gel II, Select BioProduct, USA)
- Mini-PROTEAN Electrophoresis system (Biorad)
- ChemiDoc MP system (Biorad)
- DynaMag™-2 Magnet (Thermofischer scientific, cat. no. 12321D)
- Axioscope optical microscope (Zeiss, Oberkochen, Germany) equipped with plan-neofluar objectives, UV or blue epi-illumination and differential interference contrast filters.
- Axiocam digital camera (Zeiss Oberkochen, Germany)

### Reagent setup

- **Sucrose, 2M** Dissolve 68.46 grams of sucrose in 56 mL water. Stir and heat until in solution and bring to a final volume of 100 mL. Freshly prepare the solution prior to experiment.
- **Glycine, 2M** Dissolve 15 grams of glycine in 80 mL of water. Stir until in solution and bring to a final volume of 100 mL. Store solution at 4 °C and allow solution to reach room temperature (RT) before use.
- **10X Protease Inhibitor** Dissolve cOmplete protease inhibitor, EDTA-free in 5 mL water or dissolve cOmplete protease inhibitor, mini-tablet, EDTA-free in 1 mL water. Vortex until in suspension. Freshly prepare the suspension prior to experiment. Keep at 4 °C.
- **Triton X-100, 10% (v/v)** Dissolve 5 mL of Triton X-100 in 40 mL water. Stir slowly until in solution and bring to a final volume of 50 mL. Store at Store solution at 4 °C.
- **NaCl, 5 M** Dissolve 29.22 grams of NaCl in 80 mL water. Stir until in solution and bring to a final volume of 100 mL. Autoclave and store solution at RT.
- **SDS, 10% (w/v)** Dissolve 10 grams of SDS in 80 mL water. Stir slowly and heat until in solution. Bring the solution to a final volume of 100 mL. Autoclave and store solution at RT.
- **Crosslinking buffer** contains 1% paraformaldehyde, 0.4 M sucrose, 10 mM Tris-Cl. Freshly prepare the buffer prior to experiment.
- **Buffer 1** contains 0.4 M sucrose, 10 mM Tris-Cl, 2.5% (w/v) PVP-40, 5 mM β-mercaptoethanol, 1× Roche cOmplete protease inhibitor, EDTA-free. Freshly prepare the buffer prior to experiment. Pre-chilled before use. Add β-mercaptoethanol and protease inhibitor to the buffer before use.
- **Buffer 2** contains 0.25 M sucrose, 10 mM Tris-Cl, 10 mM MgCl2, 1% (v/v) Triton X-100, 5 mM β-mercaptoethanol, 1× Roche cOmplete protease inhibitor, EDTA-free. Freshly prepare the buffer prior to experiment. Pre-chilled before use. Add β-mercaptoethanol and protease inhibitor to the buffer before use.
- **Buffer 3** contains 1.7 M sucrose, 10 mM Tris-Cl, 0.15% (v/v) Triton X-100, 5 mM β-mercaptoethanol, 1× Roche cOmplete protease inhibitor, EDTA-free. Freshly prepare the buffer prior to experiment. Pre-chilled before use. Add β-mercaptoethanol and protease inhibitor to the buffer before use.
- **Lysis buffer** contains 50 mM Tris-Cl, 10 mM EDTA, 0.1% (v/v) SDS, 1× Roche cOmplete protease inhibitor, EDTA-free. Freshly prepare the buffer prior to experiment. Pre-chilled before use. Add protease inhibitor to the buffer before use.
- **Ethanol, 70% (v/v)** add 30 mL of water into 70 mL of absolute ethanol. Prepare solution prior to experiment.
- **Tris-EDTA buffer with low EDTA (TE-lowE)** TE-lowE contains 10 mM of Tris-Cl and 0.1 mM EDTA pH.8. Store solution at 4 °C.
- **Transfer buffer** Transfer buffer contains 39 mM glycine, 48 mM tris base, 0.05%(v/v) SDS, 20% (v/v) methanol. Adjust pH to 8.3 and store at 4 °C.
- **Tris-buffered saline (TBS) 10X** Dissolve 24.23 grams of Tris base and 80.06 grams of NaCl in 800 mL water. Stir until in solution and adjust pH to 7.6 with HCl. Bring the solution to a final concentration of 1 L.
- **Tris-buffered saline with tween (TBST)** TBST contains 1X TBS, 0.5% (v/v) Tween-20. Stir slowly. Store buffer at 4 °C.
- **Blocking buffer** Dissolve 5% (w/v) non-fat milk in TBST. Stir until in suspension and keep at RT. Prepare buffer prior to experiment.
- **DAPI, 1 mg/mL** Dissolve 1 mg of DAPI dye in 1 mL water. Vortex until in solution. Keep in dark at 4 °C
